# Is level 1 trauma care necessary for all severely injured older patients? Evaluating undertriage and feasibility of care in major and non-major trauma centres in the Netherlands

**DOI:** 10.1007/s00068-025-02897-5

**Published:** 2025-06-16

**Authors:** Sara van Ameijden, Pieter Boele van Hensbroek, Doeke Boersma, Stefan van Zutphen, Martijn Poeze, Mariska de Jongh

**Affiliations:** 1 Network Emergency Care Brabant, Tilburg, The Netherlands; 2 Department of Surgery, Elisabeth-TweeSteden Ziekenhuis, Tilburg, The Netherlands; 3 Department of Surgery, Amphia Ziekenhuis, Breda, The Netherlands; 4 Department of Surgery, Jeroen Bosch Ziekenhuis, ‘s-Hertogenbosch, The Netherlands; 5 Department of Surgery, Maastricht University Medical Centre, Maastricht, The Netherlands; 6 Tilburg School of Social and Behavioral Sciences, Tilburg University, Tranzo, Tilburg, The Netherlands

**Keywords:** Aged, Multiple trauma, Mortality, Trauma centres, Triage

## Abstract

**Purpose:**

Undertriage remains a challenge within the severely injured older patients. The survival benefit in major trauma centres (MTCs) compared to non-major trauma centres (nMTCs) has been disputed. This study aimed to assess the differences in patient characteristics of severely injured older patients treated in MTCs and nMTCs and to regard whether these characteristics could be related to pre-hospital triage decisions and influence clinical outcomes in MTCs and nMTCs.

**Methods:**

A retrospective cohort study was conducted, using the Dutch National Trauma Registry to identify all patients of 70 and above with an ISS ≥ 16 during 2016–2022. Patient characteristics and outcomes between MTCs, nMTCs and directly transferred patients were compared. Backward logistic regression analyses were performed to identify factors predicting mortality.

**Results:**

A total of 10,899 patients were included. Patients in nMTCs harboured more octo- and nonagenarians than MTCs (44.6% vs. 37.2% and 15.1% vs. 6.7% resp., *p* < 0.001). The ISS was significantly lower in nMTCs (median 19 [IQR 17–25] vs. 22 [17–27], *p* < 0.001), with severe head injury and a low GCS being more prevalent in MTCs. High energy falls were more often observed in MTCs (15.6% vs. 7.7%, *p* < 0.001). Mortality was significantly lower in nMTCs (OR 0.59, 95%-CI 0.54–0.65), with a GCS 3–8 strongly associated with an increased risk for mortality in both nMTCs and MTCs (OR 19.93, *p* < 0.001 and OR 7.87, *p* < 0.001 resp.).

**Conclusion:**

The differences in patients presented in MTCs and nMTCs indicate factors contributing to undertriage; severely injured older patients with recognizable injuries and trauma mechanisms are more likely to be presented in a MTC. Whether feasible care for severely injured older patients should be provided in MTCs or nMTCs should not only be dependent on ISS and mortality rates; patient-centred care goals harbouring broader perspectives as frailty and health- and quality-of-life benefit of aggressive injury treatment should also contribute in triage- and treatment decision-making.

**Level of evidence and study type:**

Level III, prognostic/epidemiological.

## Background

The incidence of severely injured older patients increases annually, a trend observed in the Netherlands as well [1]. In the Netherlands, trauma centres are categorised in level 1, 2 or 3, depending on ED-capacity, ICU-capacity, the 24/7 availability of several specialists and several other resources. Level 1 centres are considered major trauma centres (MTCs), level 2 and 3 centres are considered non-major trauma centres (nMTCs). In order to minimize unfavourable outcomes and improve survival after severe trauma, it has been agreed upon to strive that at least 90% of the severely injured trauma patients (i.e. patients with an Injury Severity Score (ISS) above 15) should primarily be presented at MTCs [2]. However, almost one third of all severely injured patients in the Netherlands are primarily presented in nMTCs, with the proportion of undertriage increasing with age: recent findings show that of the severely injured older patients with an ISS of 16–24 and 25+, respectively 48.3% and 32.4% were primarily presented in nMTCs [1, 3].

The severely injured older patient is at a higher risk of undertriage when compared to the younger severely injured population: current triage criteria provide poor sensitivity in the older population due to altered physiological response, the presence of severe injury after relative mild trauma mechanisms, the role of polypharmacy and the higher incidence of severe mono trauma [4–6]. Furthermore, the low diagnostic accuracy for severely injured patients of the Dutch triage system, distance to a major trauma centre (MTC) and clinical judgement of Emergency Medical Services (EMS) providers contribute to the undertriage as well [7–10].

The necessity of primary presentation of severely injured older patients in a MTC has been disputed. It has been demonstrated that severely injured older patients benefit from primary presentation at a MTC, displaying lower mortality rates when compared to nMTCs [11, 12]. However, other studies showed that primary presentation at a nMTC does not consistently contributes to an increase in mortality and complications or a decrease in functional outcome [6, 13]. The above-mentioned studies do not sufficiently take the role of possible intrinsic differences between patients’ characteristics in MTCs and nMTCs, such as age, pre-existing health status and injury severity into account, whereas these characteristics might affect mortality rates and functional outcomes. Therefore, this study aims to evaluate whether patient characteristics of severely injured older patients within MTCs, nMTCs or patients directly transferred from a nMTC to a MTC differ from each other and if these differences explain pre-hospital triage decisions or impact clinical outcomes.

We hypothesize that patient characteristics from the severely injured older patients within nMTCs and MTCs intrinsically differ from each other and that these differences might indicate factors contributing to pre-hospital triage decisions, as well as influence clinical outcomes in MTCs and nMTCs. By reviewing the demographics and analysing variables that predict an increased risk of mortality in MTCs and nMTCs, we aim to identify patient groups that might benefit from earlier presentation in higher level trauma centres and to discuss what appropriate and feasible care (i.e. the extent to which care is physically, culturally and financially practical, possible and fitting within a given context [14]) for older severely injured patients comprises.

## Methods

This nationwide descriptive registry study utilised retrospective data extracted from the Dutch National Trauma Registry (DNTR). Inclusion of the data requested for this study was approved under study number LTR23.14, and the data were provided to the authors in an anonymised format. The DNTR contains data of all patients in the Netherlands who were admitted to the hospital through the emergency department (ED) within 48 h after trauma. Patients without vital signs upon arrival at the ED are not included in the DNTR database. This database contains, among other things, whether a patient is primarily presented at a MTC or nMTC, their demographics, injury mechanism, anatomical injury characteristics coded according to the Abbreviated Injury Scale (AIS, version 2005, update 2008 [15]), severity of injury according to the ISS, and diverse outcome variables as mortality, length of stay and functional outcomes [16]. The ISS is calculated as a derivative of AIS, defined as the sum of the squares of the highest AIS grade in each of the three most severely injured areas [17].

### Population

This study included all patients in the DNTR database from 2016 to 2022, aged 70 years and older at the time of initial presentation and were classified as severely injured. Patients were divided in cohorts according to primary ED-presentation at either MTCs and nMTCs. Additionally, the cohort which primarily presented at a nMTC but was directly transferred to a MTC was analysed as well. It should be noted that these patients are embedded in both the MTC and nMTC cohort. Age was stratified into cohorts of 10 years: 70–79, 80–89, and ≥ 90 years. The reported American Society of Anaesthesiologists (ASA) score is based on the pre-injury condition of the patient.

ISS was dichotomized into ‘severely injured (ISS 16–24)’ and ‘very severely injured (ISS ≥ 25)’ [18]. The injury characteristics for each body region were classified by the AIS, and very severe injury (AIS ≥ 4) of each body region (head, neck, spine, thorax, abdomen, upper extremities, lower extremities and external) was reported. Regarding injury mechanisms, the study differentiated between traffic incidents (pedestrian, bicycle, motorised vehicle, other), low-energy falls (< 3 times body height), high-energy falls (≥ 3 times body height), and other mechanisms (gunshot and knife injuries, blunt object injuries, burn injuries, and other mechanisms not covered by the aforementioned categories).

### Outcome

Hospital mortality was analysed as primary outcome in MTCs and nMTCs. Additionally, 30-day mortality, functional outcome, length of hospital stay (LOS), Intensive Care Unit (ICU) admission and discharge destination were analysed as secondary outcomes. Functional outcome was reported using the Glasgow Outcome Scale (GOS). It should be noted that outcome variables for the directly transferred patients could not be analysed separately, since the outcome variables of the directly transferred patients are grouped with all patients that were admitted to a MTC and cannot be derived from the DNTR.

### Statistical analysis

Nominal and categorical data were presented as quantity and percentage, continuous data as mean and standard deviation (SD) or as median and inter-quartile range (IQR) depending on the distribution. Dichotomous and categorical variables were tested using the Pearson chi-square test or Fisher’s exact test, continuous variables were tested using the Kruskal-Wallis H-test due to the non-parametric distribution. All p-values smaller than 0.05 were considered statistically significant.

To identify patient characteristics predictive of an increased risk on mortality, first, characteristics were tested with univariate analyses. Variables that showed a significantly odds ratio on mortality were then entered in a stepwise, backwards logistic regression analysis for both MTCs and nMTCs. The variables age, gender, ASA, ISS and Glasgow Coma Score (GCS) were analysed in both backward models; only complete case analysis was performed. Variables with a p-value > 0.10 were removed from the model with each step [19]. Performance of the analyses were assessed based on goodness-of-fit, discrimination and percentage of variability, using Hosmer-Lemeshow test, area under the ROC curve (AUC) and Nagelkerke R² respectively. An AUC under 0.5 suggests no discrimination, 0.5 to 0.7 is considered acceptable, 0.8 to 0.9 excellent and an AUC above 0.9 considered outstanding [20].

All statistical analyses were performed using IBM SPSS Statistics version 24.0 and R version 3.6.0 (2019-04−26).

## Results

### Patient characteristics

A total of 10,899 patients were included in our cohort: 6,321 were primarily presented in a MTC, 4,578 in a nMTC, of which 458 were directly transferred to a MTC. Patients in the nMTC cohort harboured a significantly larger proportion of nonagenarians (90+) in comparison with the MTC and directly transferred cohort (13.9% vs. 6.7% and 2.8% respectively, *p* < 0.001), whereas the directly transferred patients were significantly younger than the MTC and nMTC cohort (mean 77.9 years vs. 79.1 and 81.3, *p* < 0.001).

The distribution of severely and very severely injured older patients in MTC and directly transferred patients were comparable. However, in the nMTC cohort, the proportion significantly shifted in favour of the severely injured (ISS 16–24) when compared to the MTC and directly transferred patients. In all cohorts, trauma was most commonly caused by low energetic falls, with these falls being more prevalent in nMTCs than in MTCs (57.0% vs. 42.4%, *p* < 0.001). In MTCs however, bicycle accidents and high-energy falls were more common. The most affected body region was the head, with very severe head trauma being more prevalent in MTCs and directly transferred patients than in nMTCs (51.3% and 53.1% vs. 45.2%, *p* < 0.001). Even though the prevalence of severe head trauma was comparable between MTCs and directly transferred patients, this was not reflected in the GCS at arrival. Whereas 28.2% of the MTC cohort presented with a GCS between 3 and 8, only 7.5% of the directly transferred patients did so (Table 1).

**Table 1:** Patient characteristics

	All patients	Primarily presented in MTCs	Primarily presented in nMTCs (including directly transferred to MTC)	Primarily presented in nMTCs, directly transferred to MTCs
	*n* = 10,899	*n* = 6,321	*n* = 4,578	*n* = 458
**Age (years) Mean - SD**	80.0 (±6.7)	79.1 (±6.4)	81.3 (±7.0)	77.9 (±5.7)
70-79	5494 (50.4%)	3543 (56.1%)	1951 (42.6%)	293 (64.0%)
80-89	4346 (39.9%)	2354 (37.2%)	1992 (43.5%)	152 (33.2%)
90+	1059 (9.7%)	424 (6.7%)	635 (13.9%)	13 (2.8%)
**Sex**				
Male	5819 (53.4%)	3556 (56.3%)	2263 (49.4%)	261 (57.0%)
Female	5078 (46.6%)	2763 (43.7%)	2315 (50.6%)	197 (43.0%)
**Injury Severity Scale**				
Median - IQR	21 [17-26]	22 [17-27]	19 [17-25]	22 [17-26]
Category 16-24	6572 (60.3%)	3397 (53.7%)	3175 (69.4%)	257 (56.1%)
Category 25-75	4327 (39.7%)	2924 (46.3%)	1403 (30.6%)	201 (43.9%)
**Injury mechanism**				
Traffic				
Bicycle	2049 (19.3%)	1418 (22.9%)	631 (14.2%)	86 (19.1%)
Motorised	788 (7.4%)	558 (9.0%)	230 (5.2%)	39 (8.6%)
Pedestrian	299 (2.8%)	227 (3.7%)	72 (1.6%)	16 (3.5%)
Other	80 (0.8%)	49 (0.8%)	31 (0.7%)	1 (0.2%)
Fall LET	5160 (48.5%)	2627 (42.4%)	2533 (57.0%)	220 (48.8%)
Fall HET	1312 (12.3%)	964 (15.6%)	348 (7.8%)	44 (9.8%)
Other	518 (4.9%)	317 (5.7%)	201 (4.5%)	23 (5.1%)
Missing	431 (4.1%)	37 (0.6%)	394 (8.9%)	22 (4.9%)
**Injuries**				
≥ 2 injuries reported	10,017 (91.9%)	5965 (94.4%)	4052 (88.5%)	404 (88.2%)
Head AIS =>4 (n, %)	5314 (48.8%)	3243 (51.3%)	2071 (45.2%)	243 (53.1%)
Spine AIS=>4 (n, %)	636 (5.8%)	470 (7.4%)	166 (3.6%)	44 (9.6%)
Abdomen AIS=>4 (n, %)	277 (2.5%)	149 (2.4%)	128 (2.8%)	14 (3.1%)
Thorax AIS=>4 (n, %)	1250 (11.5%)	755 (11.9%)	495 (10.8%)	33 (7.1%)
Lower extremities AIS=>4 (n, %)	445 (4.1%)	255 (4.0%)	190 (4.2%)	37 (8.1%
External AIS=>4 (n, %)	251 (2.3%)	146 (2.3%)	105 (2.3%)	4 (0.9%)
**ASA (n, valid %)**				
ASA I	849 (8.7%)	590 (10.3%)	259 (6.5%)	45 (10.8%)
ASA II	5056 (51.7%)	3019 (52.6%)	2037 (51.1%)	228 (54.8%)
ASA III	3532 (36.1%)	1984 (34.5%)	1548 (38.9%)	139 (33.4%)
ASA IV	270 (2.8%)	136 (2.4%)	134 (3.4%)	4 (1.0%)
ASA V	22 (0.2%)	16 (0.3%)	6 (0.2%)	0 (0.0%)
Missing	1119	576	543	42
**GCS (n, valid %)**				
3	1263 (12.9%)	1123 (20.2%)	131 (3.1%)	8 (1.9%)
4-5	197 (2.0%)	130 (2.3%)	67 (1.6%)	3 (0.7%)
6-8	450 (4.6%)	331 (5.9%)	119 (2.8%)	15 (3.6%)
9-12	926 (9.5%)	661 (11.9%)	265 (6.3%)	40 (9.5%)
13-15	6928 (71.0%)	3327 (59.7%)	3601 (86.1%)	355 (84.3%)
Missing	1144	749	395	37

Abbreviations: MTC: major trauma centre; nMTC: non-major trauma centre; ASA: American Society of Anaesthesiologists; GCS: Glasgow Coma Score

All variables were tested using the Chi-square test, unless stated otherwise. Body regions that were affected in less than 1% of the population (i.e. neck, upper extremities) were not visualised in this table

### Clinical outcomes

Patients primarily presented in a MTC showed significantly higher hospital mortality (33.1% vs. 22.7%, *p* < 0.001) and 30-day mortality (39.3% vs. 30.1%, *p* < 0.001) when compared to patients primarily presented at a nMTC. The MTC cohort was significantly more often admitted to the ICU (56.0% vs. 26.5%, *p* < 0.001) and posed for a longer hospital length of stay (median 10 (IQR 5–17) vs. 8 (IQR 4–14) days, *p* < 0.001). After discharge, the nMTC cohort could more often return to their pre-existent living condition, whereas the MTC cohort were more often discharged to another hospital (e.g. the hospital they originally were presented at) or care institute (Table 2).

**Table 2:** Clinical outcomes in MTCs and nMTCs

*N* = 10,431	MTC	nMTC*	*p*-value
	*N* = 6,321	*N* = 4,100	
**Hospital mortality (n, %)**			# *p* < 0.001
Survivors	4227 (66.9%)	3169 (77.3%)	
Non-survivors	2093 (33.1%)	931 (22.7%)	
Missing	1	0	
**30-day mortality (n, %)**			# *p* < 0.001
Survivors	3321 (58.1%)	2451 (66.1%)	
Non-survivors	2246 (39.3%)	1115 (30.1%)	
Missing	607	290	
**ICU admission %**	3448 (56.0%)	1065 (26.5%)	# *p* < 0.001
Length of hospital stay**			¥ *p* < 0.001
(median-IQR)	10 (5–17)	8 (4–14)	
Glasgow Outcome Scale** (%)			# *p* <0.001
Good recovery	552 (13.8%)	503 (17.5%)	
Moderate disability	2185 (54.5%)	1558 (45.2%)	
Severe disability	871 (21.7%)	493 (17.2%)	
Persistent vegetative state	37 (0.9%)	26 (0.9%)	
Missing	367	292	

*excluding directly transferred patients ** excluding deceased patients

# Chi-square Test, ¥ Kruskal Wallis H Test

Abbreviations: MTC: major trauma centre; nMTC: non-major trauma centre; ICU: Intensive Care Unit

### Identifying patient characteristics predictive for mortality

All included variables showed p-values < 0.10 in the first step of the analyses. Therefore, no variables were excluded stepwise from the analyses.

In both MTCs and nMTCs, GCS 3–8 was the strongest predictor of in-hospital mortality, with 7.9 more odds in MTCs, and up to 19.9 more odds for patients primarily presented in a nMTC.

Additionally, high ASA scores were associated with an increased risk for mortality in both MTCs and nMTCs as well, especially in nMTCs. As portrayed in Table 3, both an ISS of 25 and above and increased age were significant predictors for in-hospital mortality, especially in MTCs.

Both models displayed excellent discrimination, portraying an AUC of 0.83 (95% CI 0.820–0.841) for the MTC model and an AUC of 0.83 (95% CI 0.820–0.840) for the nMTC model. The variability was moderately explained within both models, Nagelkerke’s R^2^ being 0.414 and 0.351 respectively. Goodness-of-fit for the nMTC model was not significant (*p* = 0.067), resulting in an adequate fit. However, the fit for the MTC-model was poor, displaying high significance (*p* < 0.001).

**Table 3:** Multivariable analyses on mortality for MTC and nMTC

	MTC			nMTC*		
	OR	*P*	95% CI	OR	*P*	95% CI
**Age (years)**						
*70–79*	*ref*			*ref*		
*80–89*	1.96	<0.001	1.67–2.29	2.19	<0.001	1.74–2.76
*90+*	4.08	<0.001	3.07–5.44	2.59	<0.001	1.92–3.49
**Sex Female**	0.80	0.003	0.69–0.92	0.67	<0.001	0.55–0.82
**Comorbidities**						
*ASA 1*	*ref*			*ref*		
*ASA 2*	1.1	0.205	0.91–1.57	1.41	0.23	0.80–2.48
*ASA 3*	1.81	<0.001	1.37–2.41	2.49	0.002	1.40–4.40
*ASA 4*	5.00	<0.001	3.08–8.14	7.64	<0.001	3.90–15.00
**ISS 25+**	4.45	<0.001	3.83–5.17	3.71	<0.001	3.05–4.52
**GCS 3–8**	7.87	<0.001	6.72–9.21	19.93	<0.001	13.58–29.23

*excluding directly transferred patients. Abbreviations: MTC: major trauma centre; nMTC: non-major trauma centre; ASA: American Society of Anaesthesiologists; GCS: Glasgow Coma Score. Only complete case analysis was performed. For MTCs, 5,024 cases were included. For nMTCs, 3,324 cases were included. ASA 5 was analysed, but not visualised in this table due to the limited number of cases (*n* < 10)

**Fig. 1:**
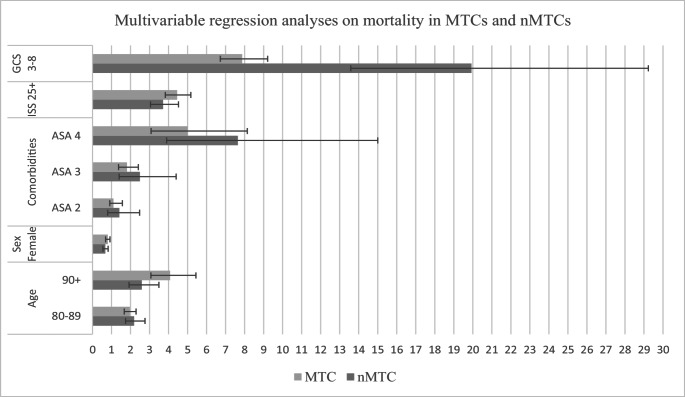
Visual representation multivariable analyses on mortality in MTCs and nMTCs

## Discussion

This study highlights the intrinsic differences between patient characteristics of the severely injured older trauma patients primarily presented in MTCs, nMTCs and directly transferred patients. These differences might indicate factors contributing to pre-hospital triage decisions and influence clinical outcomes in MTCs and nMTCs. Older trauma patients in MTCs and directly transferred patients are relatively younger and in better pre-existing condition, but more severely injured than the older patients in nMTCs, posing for higher in-hospital mortality rates and ICU admissions in MTCs. A GCS 3–8 at arrival was very strongly associated with mortality in nMTCs and, in a lesser extent, with mortality in MTCs. These results highlight the multiple, patient-individual factors contributing to the decision where final treatment for the older severely injured patient should take place.

In the Netherlands, the national guideline for field triage includes, among other things, vital signs, injury characteristics and injury mechanisms to accurately estimate injury severity [21]. Injury characteristics that are easily recognised as severe, such as visual deformities, multiple injured body regions or the presence of neurological symptoms, are associated with a lower risk of undertriage [22, 23]. In this study, the nMTC cohort showed a higher proportion of ISS 16–24. It is hypothesized that an ISS between 16 and 24 is often comprised of a combination of “less severe” injuries (e.g. older patients primarily presenting with hip fractures, in combination with concomitant injuries such as rib fractures or mild traumatic brain injury (TBI) being identified after additional imaging), leading to a decreased recognizability of severity before additional imaging has taken place. Additionally, injuries causing neurological deficits were more prevalent in the MTC cohort: severe injury of the spine was twice as prevalent in MTCs when compared to nMTCs, possibly caused by the accompanying neurological symptoms which makes the severity of those injuries more recognizable. This also applies to the prevalence of severe TBI in combination with a low GCS in MTCs: a low GCS is easily recognized and associated with a lower risk of undertriage [23]. However, optimally triaging those with severe TBI and an initially good consciousness in the pre-hospital setting remains challenging, leading to a higher risk of undertriage [22]. A good initial GCS does not rule out the presence of severe or progressive TBI, which was highlighted within the directly transferred patients: even though the prevalence of severe TBI was high (over 50%), this was not directly reflected in a low GCS. The severity of TBI based upon the GCS might therefore be underestimated before imaging had taken place, contributing to possible undertriage (and possible delay of MTC care) in this subgroup.

The trauma mechanism attributes to recognizability of trauma severity as well. In patients primarily presented in nMTCs, injuries were more often caused by low-energy falls, whereas traffic accidents and high-energy falls were more prevalent in MTCs. Since relatively mild trauma mechanisms can cause severe injuries within the older population, the severity of sustained trauma is more likely to be underestimated after low-energy mechanisms, therefore influencing pre-hospital triage decisions [4, 24, 25].

In order to minimize undertriage within the severely injured older patients, geriatric specific triage tools have been developed. However, current elderly specific triage criteria are variable, focus on different physiologic thresholds and lack consensus on the optimal triage tool [5, 26–28]. Strategies to improve geriatric specific triage should consider the development of validated geriatric specific variables and thresholds based on costs, benefits and patient preferences [25]. However, given the current overall limited performance of used triage models on individual severely injured older patients, pre-hospital health care providers should consider primary presentation at a MTC with a low threshold, keeping in mind that severe injury might be underestimated within this population.

The intrinsic differences in sustained injuries and subsequent ISS pose for a challenge when directly comparing outcomes in MTCs and nMTCs. The higher mortality rate and percentage of ICU admission in MTCs can be explained by the higher proportion of very severely injured patients within MTCs. Even though the mortality rate of severely injured older patients is significantly higher in comparison with younger age categories [29], mortality rates might not be the only appropriate outcome variable to analyse in order to determine whether MTC care is more feasible and appropriate for severely injured older patient than nMTC care. A specific subgroup to highlight this issue with are the older patients with severe TBI, and especially those with an additional low GCS. This study showed that in nMTCs, a low GCS was associated with a 20-fold increased risk for mortality. Survival benefit for patients with severe TBI when transferred to a MTC, regardless of the additional time to definitive care, has been described [30], suggesting that some of these severely injured older patients might benefit from a higher degree of transferral or earlier primary presentation at a MTC.

However, it could also be hypothesized that within older patients presented in a nMTC, severe TBI accompanied by a low GCS is sometimes serious to the extent that the chance of survival (or the chance of acceptable quality of life in case of survival) is deemed minimal. Subsequently, transferral to a MTC might not be deemed feasible after careful consideration of health care providers, the patient and their family. For the older trauma patient with severe TBI and a negligible chance of survival or recovery, withdrawal of life-sustainable therapy (WLST) can be considered [31]. One should consider whether initial presentation or transferral to a MTC, often further away from family, for these patients is futile [25]. Detailed patient file analysis is needed in order to determine the prevalence and effect of WLST on hospital mortality of the severely injured older patients in both MTCS and nMTCs.

Demographic characteristics such as advanced age and pre-existent comorbidities are relevant as well when considering appropriate care at the right place for the severely injured older patient. Patients primarily presented at nMTCs were significantly older than the MTC cohort, with an even bigger difference identified when compared to the directly transferred cohort. Patients in MTCs and directly transferred patients were in better pre-existent condition, portraying lower pre-injury ASA-scores. In both MTCs and nMTCs, higher age was associated with a significant increased risk for mortality. A higher ASA score significantly increased the risk for mortality, especially in nMTCs, even more than high age did. Therefore, the survival benefit of aggressive injury treatment when age and the number or severity of comorbidities increases should be discussed. This study demonstrated that patients were selected for direct transferral to MTCs when they displayed characteristics that were associated with a lower OR on mortality: directly transferred patients were significantly younger and had fewer pre-existing comorbidities than patients receiving definite care at nMTCs. This indicates that age and comorbidities are of influence when considering the feasibility of primary presentation at or a transfer to a higher-level trauma centre [10, 30, 32], and might affect the decision where final treatment of the severely injured older patient should take place.

However, there should be caution when deciding upon a less aggressive treatment for this population: given their limited physiological reserves, older severely injured patients might benefit from earlier aggressive treatment within their window of opportunity [33]. Even at exceedingly high ISS scores, no combination of ISS, age and comorbidities demonstrated a mortality rate deemed as futile (i.e. 95%) [34], underlining that older severely injured trauma patients should not per se be excluded from aggressive injury treatment based on age or comorbidities alone [12, 31]. However, the significant higher proportion of octo- and nonagenarians with higher ASA scores in nMTCs suggests the possibility that some older severely injured patients might receive lower level trauma care due to their age or pre-existent health condition, and that these patients should be primarily presented to or be transferred to MTCs with a lower threshold.

The used data does not give any insight into whether the benefits of transferral, (neuro)surgical options or aggressive treatment for individual patients were discussed between healthcare providers in nMTCs and MTCs, or with patients and family members. Detailed patient file analysis is needed in order to determine whether the benefit of transferral to a MTC is discussed between different levels of trauma centres, and which patients are consciously kept at nMTCs because the right care for an individual patient can be provided in nMTCs as well.

Discussing the benefit of and whether or not aggressive trauma treatment is preferred with patients and their family underlines the relevance of the older patient’s attitude towards appropriate and feasible care. In the Netherlands, Advanced Care Planning (ACP) and treatment preferences are often discussed between geriatric patient and their general practitioners [35], with at least one preference regarding WLST known in 45% of the geriatric patients referred to the ED [36]. When a patient has documented that they do not wish to undergo life-sustaining treatments, or do not wish to receive ICU care, the necessity of transferral to a MTC after sustaining severe injury can be questioned. Within the current database, no conclusion can be drawn to which extent ACP wishes influence triage- and transferral-decision making.

When determining what the right care at the right place for which severely injured older patients is, this study highlighted the complex interplay between possible contributors to pre-hospital triage decisions, the ensuing differences between MTC, nMTC and directly transferred cohorts and how these differences influence hospital mortality and the definition of feasible care for this population. Given the often complexity in patient treatment that comes with high age, it should be considered whether the challenge of ‘right care at the right place’ should revolve around centres specialised on geriatric trauma care, instead of ISS and subsequent MTC versus nMTC care. Independent of trauma centre performance, older trauma patients are less likely to die when presented at a centres with a high geriatric case volume [37, 38], underscoring the importance of this debate.

### Limitations

This study has several limitations. Firstly, due to the coding of directly transferred patients in the DNTR, the demographics and clinical outcomes of these patients are integrated within the MTC cohort. As stated within the method, the directly transferred patients are both shown as a separate group, but are also embedded within the entire group of MTC patients. Therefore, demographic data of MTC patients might be slightly skewed. When analysing outcome variables, the directly transferred patients are only portrayed in the MTC cohort. Since directly transferred patients receive their final treatment in MTCs, the authors do not expect that this causes bias of the clinical outcomes in MTCs. The directly transferred patients were excluded from the clinical outcomes of the nMTCs, to prevent skewing of these outcomes.

Secondly, the DNTR itself is limited when considering the effect of several variables on pre-hospital decision-making and mortality within this often complex population. For instance, the effect of specific comorbidities or frailty (instead of the documented ASA), patient-reported ACP-preferences and referral considerations, as well as the effect of pre-hospital GCS scores cannot be analysed. Future studies should document these factors as well, in order to gain a more detailed insight of additional factors that determine feasible care for the severely injured older patients.

Lastly, the multivariable analyses in this study, although explaining the variance in both MTCs and nMTCs for 30–40%, provide a poor goodness-of-fit. This underlines that several factors attributing to mortality in MTCs and nMTCs were not identified in this study, and might be embedded within the limited knowledge about the prevalence of specific comorbidities or frailty.

## Conclusion

Significant differences in age, pre-existing comorbidities and injury characteristics are observed between severely injured older patients primarily presented in MTCs, nMTCs and directly transferred patients. These differences are partially found in factors which might be related to undertriage; when injuries and trauma mechanisms are easily recognized as severe, a severely injured older patient is more likely to be primarily presented in a MTC. Especially the recognizability of severe TBI with an initial good GCS remains challenging; these patients might benefit from earlier primary presentation at or transferral to a MTC, given the high risk for mortality when GCS deteriorates. Furthermore, age, pre-existent comorbidities and possible known ACP-wishes should be considered individually in triage decision-making, but should not be an overall barrier for treatment in a MTC.

Whether feasible care for severely injured older patients should be provided in MTCs or nMTCs should not only be dependent on ISS and mortality rates, but should take broader perspectives such as frailty, ACP preferences and the benefit of aggressive injury treatment into account as well. Future research should focus on how these patient-centred care goals affect (under)triage, mortality and the definition of feasible care for the older severely injured trauma patient.

## Data Availability

This study used data from the Dutch National Trauma Registry; inclusion of the data requested for this study was approved under study number LTR23.14, and the data were provided to the authors in an anonymised format. Data requests of the Dutch National Trauma Registry can be submitted to the Landelijk Netwerk Acute Zorg (LNAZ).

## References

[1] van Ameijden SI, De Jongh MAC, Poeze M. The severely injured older patient: identifying patients at high risk for mortality using the Dutch National trauma registry. Eur J Trauma Emerg Surg. 2024. 10.1007/s00068-024-02738-x 10.1007/s00068-024-02738-x PMC11761987 39856260

[2] National Health Care Institute. Spoed moet goed: indicatoren en normen voor zes spoedzorgindicaties. 2015.

[3] Sturms LM, Driessen MLS, van Klaveren D, Ten Duis H, Kommer G, Bloemers FW, et al. Dutch trauma system performance: are injured patients treated at the right place? Injury. 2021;52(7):1688–96. 10.1016/j.injury.2021.05.015. 34045042 10.1016/j.injury.2021.05.015

[4] Hoyle AC, Biant LC, Young M. Undertriage of the elderly major trauma patient continues in major trauma centre care: a retrospective cohort review. Emerg Med J. 2020;37(8):508–14. 10.1136/emermed-2019-208541. 32546474 10.1136/emermed-2019-208541

[5] Alshibani A, Singler B, Conroy S. Towards improving prehospital triage for older trauma patients. Z Gerontol Geriatr. 2021;54(2):125–9. 10.1007/s00391-021-01844-4. 33507358 10.1007/s00391-021-01844-4

[6] Fröhlich M, Casper M, Lefering R, Driessen A, Bouillon B, Maegele M, et al. Do elderly trauma patients receive the required treatment? Epidemiology and outcome of geriatric trauma patients treated at different levels of trauma care. Eur J Trauma Emerg Surg. 2020;46(6):1463–69. 10.1007/s00068-019-01285-0. 31844920 10.1007/s00068-019-01285-0

[7] van Laarhoven JJEM, Lansink KWW, van Heijl M, Lichtveld RAA, Leenen LPH. Accuracy of the field triage protocol in selecting severely injured patients after high energy trauma. Injury. 2014;45(5):869–73. 10.1016/j.injury.2013.12.010. 24472800 10.1016/j.injury.2013.12.010

[8] Voskens FJ, van Rein EAJ, van der Sluijs R, Houwert RM, Lichtveld RA, Verleisdonk EJ, et al. Accuracy of prehospital triage in selecting severely injured trauma patients. JAMA Surg. 2018;153(4):322–7. 10.1001/jamasurg.2017.4472. 29094144 10.1001/jamasurg.2017.4472 PMC5933379

[9] Waalwijk JF, Lokerman RD, van der Sluijs R, Fiddelers AAA, Leenen LPH, Poeze M, et al. Evaluating the effect of driving distance to the nearest higher level trauma centre on undertriage: a cohort study. Emerg Med J. 2022;39(6):457–62. 10.1136/emermed-2021-211635. 34593562 10.1136/emermed-2021-211635

[10] Chang DC, Bass RR, Cornwell EE, MacKenzie EJ. Undertriage of elderly trauma patients to state-designated trauma centers. JAMA Surg. 2008;143(8):776–81. 10.1001/archsurg.143.8.776. 10.1001/archsurg.143.8.776 18711038

[11] El-Qawaqzeh K, Magnotti LJ, Hosseinpour H, Nelson A, Spencer AL, Anand T, et al. Geriatric trauma, frailty, and ACS trauma center verification level: are there any correlations with outcomes? Injury. 2023;7. 10.1016/j.injury.2023.110972. 10.1016/j.injury.2023.110972 37573210

[12] Garwe T, Stewart K, Newgard CD, Stoner J, Sacra JC, Cody P, et al. Survival benefit of treatment at or transfer to a tertiary trauma center among injured older adults. Prehosp Emerg Care. 2020;24(2):245–56. 10.1080/10903127.2019.1632997. 31211622 10.1080/10903127.2019.1632997 PMC6962564

[13] Rogers FB, Morgan ME, Brown CT, Vernon TM, Bresz KE, Cook AD, et al. Geriatric trauma mortality: does trauma center level matter? Am Surg. 2021;87(12):1965–71. 10.1177/0003134820983190. 10.1177/0003134820983190 33382347

[14] Pearson A, Wiechula R, Court A, Lockwood C. The JBI model of evidence-based healthcare. Int J Evid Based Healthc. 2005;3(8):207–15. 10.1111/j.1479-6988.2005.00026.x. 21631749 10.1111/j.1479-6988.2005.00026.x

[15] Association for the Advancement of Autmotive Medicine. Abbreviated Injury Scale. 2008.

[16] Landelijk Netwerk Acute Zorg (LNAZ). Dutch National Trauma Registry (DNTR) 2007. Available from: https://www.lnaz.nl/trauma/landelijke-traumaregistratie

[17] Baker SP, O’Neill B, Haddon WJ, Long WB. The injury severity score: a method for describing patients with multiple injuries and evaluating emergency care. J Trauma. 1974;14(3):187–96. 4814394

[18] Champion HR, Copes WS, Sacco WJ, Lawnick MM, Keast SL, Frey CF. The major trauma outcome study: establishing national norms for trauma care. J Trauma. 1990;30(11):1356–65. 10.1097/00005373-199011000-00008. 2231804

[19] Twisk JWR. Inleiding in de toegepaste Biostatistiek. Amsterdam: Reed Business; 2011.

[20] Hosmer DW Jr, Lemeshow S. Applied Logistic Regression: Wiley; 2000.

[21] Ambulancezorg Nederland. Landelijk Protocol Ambulancezorg Nederland versie 9 (LPA9). 2022.

[22] Shinji N, Tetsuya M, Masato U, Yasuaki M, Masao I, Junichiro Y, Katsumi Y. Predictive factors for undertriage among severe blunt trauma patients: what enables them to slip through an established trauma triage protocol? J Trauma. 2010;68(5):1044–51. 10.1097/TA.0b013e3181aca144. 10.1097/ta.0b013e3181aca144 20480540

[23] Amoako J, Evans S, Brown NV, Khaliqdina S, Caterino JM. Identifying predictors of undertriage in injured older adults after implementation of statewide geriatric trauma triage criteria. Acad Emerg Med. 2019;26(6):648–56. 10.1111/acem.13695. 30661273 10.1111/acem.13695

[24] van Rein E, Sadiqi S, Lansink KWW, Lichtveld RA. The role of emergency medical service providers in the decision-making process of prehospital trauma triage. Eur J Trauma Emerg Surg. 2020;46(1). 10.1007/s00068-018-1006-8. 10.1007/s00068-018-1006-8 PMC7026224 30238385

[25] Fuller G, Pandor A, Essat M, Sabir L, Buckley-Woods H, Chatha H, et al. Diagnostic accuracy of prehospital triage tools for identifying major trauma in elderly injured patients: a systematic review. J Trauma Acute Care Surg. 2021;90(2):403–12. 10.1097/TA.0000000000003039. 33502151 10.1097/TA.0000000000003039

[26] Boulton AJ, Peel D, Rahman U, Cole E. Evaluation of elderly specific pre-hospital trauma triage criteria: a systematic review. Scand J Trauma Resusc Emerg Med. 2021;29(1):127. 10.1186/s13049-021-00940-z. 34461976 10.1186/s13049-021-00940-z PMC8404299

[27] Pandor A, Fuller G, Essat M, Sabir L, Holt C, Woods HB, Chatha H. Individual risk factors predictive of major trauma in pre-hospital injured older patients: a systematic review. Br Paramed J. 2022;6(4):26–40. 10.29045/14784726.2022.03.6.4.26. 35340581 10.29045/14784726.2022.03.6.4.26 PMC8892449

[28] Werman HA, Erskine T, Caterino J, Riebe JF, Valasek T, Members of the Trauma Committee of the State of Ohio EMS Board. Development of statewide geriatric patients trauma triage criteria. Prehosp Disaster Med. 2011;26(3):170–9. 10.1017/S1049023X11006315. 22107767 10.1017/S1049023X11006315

[29] De Vries R, Reininga IHF, Pieske O, Lefering R, El Moumni M, Wendt KW. Injury mechanisms, patterns and outcomes of older polytrauma patients—an analysis of the Dutch trauma registry. PLoS ONE. 2018;13(1). 10.1371/journal.pone.0190587. 10.1371/journal.pone.0190587 PMC5755835 29304054

[30] Waalwijk JF, Lokerman RD, van der Sluijs R, Fiddelers AAA, den Hartog D, LLP H, et al. The influence of inter-hospital transfers on mortality in severely injured patients. Eur J Trauma Emerg Surg. 2023;49(1):441–9. 10.1007/s00068-022-02087-7. 36048180 10.1007/s00068-022-02087-7 PMC9925487

[31] van Wessem KJP, Leenen LPH. Geriatric polytrauma patients should not be excluded from aggressive injury treatment based on age alone. Eur J Trauma Emerg Surg. 2022;48(1):357–65. 10.1007/s00068-020-01567-y. 33320284 10.1007/s00068-020-01567-y PMC7736672

[32] Garwe T, Cowan LD, Neas B, Cathey T, Danford BC, Greenawalt P. Survival benefit of transfer to tertiary trauma centers for major trauma patients initially presenting to nontertiary trauma centers. Acad Emerg Med. 2010;17(11):1223–32. 10.1111/j.1553-2712.2010.00918.x. 21175521 10.1111/j.1553-2712.2010.00918.x

[33] Spering C, Lefering R, Bouillon B, Lehmann W, von Eckardstein K, Dresing K, Sehmisch S. It is time for a change in the management of elderly severely injured patients! An analysis of 126,015 patients from the traumaregister DGU^®^. Eur J Trauma Emerg Surg. 2020;46:487–97. 10.1007/s00068-019-01229-8. 31520156 10.1007/s00068-019-01229-8

[34] Duvall DB, Zhu X, Elliott AC, Wolf SE, Rhodes RL, Paulk MA, Phelan HA. Injury severity and comorbidities alone do not predict futility of care after geriatric trauma. J Palliat Med. 2015;18(1):246–50. 10.1089/jpm.2014.0336. 25494453 10.1089/jpm.2014.0336 PMC4347887

[35] van der Steen J, Engels Y, Touwen DP, Kars MC, Reyners AKL, van der Linden YM, Korfage IJ. Advance care planning in the Netherlands. Zeitschrift Für Evidenz Fortbildung und Qualität im Gesundheitswesen. 2023;180:133–8. 10.1016/j.zefq.2023.06.003. 37482528 10.1016/j.zefq.2023.06.003

[36] Ermers DJM, van Beuningen-van Wijk MPH, Peters Rit E, Stalpers-Konijnenburg SC, Taekema DG, Bosch FH, et al. Life-sustaining treatment preferences in older patients when referred to the emergency department for acute geriatric assessment: a descriptive study in a Dutch hospital. BMC Geriatr. 2021;21(58). 10.1186/s12877-020-02002-y. 10.1186/s12877-020-02002-y PMC7807792 33446116

[37] Zafar SN, Obirieze A, Schneider EB, Hashmi Z, Scott V, Greene W, et al. Outcomes of trauma care at centers treating a higher proportion of older patients. The case for geriatric trauma centers. J Trauma Acute Care Surg. 2015;78(4):852–9. 10.1097/TA.0000000000000557. 25742246 10.1097/TA.0000000000000557

[38] Kojima M, Endo A, Zakhary B, Shoko T, Firek M, Coimbra R. Case volume and rate are associated with outcomes in geriatric trauma: a case for geriatric trauma centers? J Trauma Acute Care Surg. 2023;94(2):241–7. 10.1097/TA.0000000000003838. 36399493 10.1097/TA.0000000000003838

